# 

**Published:** 1885-06

**Authors:** 


### EDITORIAL.

THE Twenty-Third Annual Meeting of the Iowa State Dental Society was held in Des Moines, Tuesday, May 5th, continuing four days. The attendance was the largest the society ever had. The papers and discussions and the clinics were very interesting and instructive. The membership was increased by twenty-seven. The society will publish its transactions in full in pamphlet form.

The officers elected are as follows: President, A. Morsman, Iowa City; Vice-President, R. L. Cochran, Burlington; Secretary, J. B. Monfort, Fairfield; Treasurer, J. S. Kulp, Muscatine.

Society adjourned to meet in Iowa City, the first Tuesday in May, 1886. J. B. MONFORT, Secretary.

### Indiana State Dental Association.

The Twenty-Seventh Annual Meeting of the Indiana State Dental Association will be held at Lake Maxinkuckee, Tuesday, June 30th, 1885. The State Board of Dental Examiners will also meet at the same time and place.

R. W. VAN VALZAH, Secretary,
Terre Haute, Ind.

We trust that the above notice will receive the attention its importance deserves. This is one of the oldest of our State societies. Its meetings have always been conducted with marked ability. It has in its membership some of the best men of the dental profession, men whom to meet is a pleasure and a stimulus to higher attainment.

Maxinkuckee, is a delightful place to spend a few days, and as compared with a city with its turmoil and bustle, is a most agreeable relief.

It is confidently expected that this will be a large and interesting meeting.

### CONTENTS.

### PROCEEDINGS.

Southern Dental Association .......... 265

SALMAGUNDI .......... 327

### EDITORIAL.

Indiana State Dental Association .......... 334

Southern Dental Association .......... 335

Dissolution .......... 335

Obituary .......... 335

---

### THE DENTAL REGISTER,

A MONTHLY MAGAZINE DEVOTED TO THE INTERESTS OF THE

### DENTAL PROFESSION.

*Terms Reduced to* $2.00 *Per Year. Single Copies,* 25 *Cents.*

**EDITED BY PROF. J. TAFT, D.D.S.**

—AND—

**Published by SPENCER & CROCKER, Ohio Dental and Surgical Depot.**

The volume commences in January and closes in December.

The REGISTER being issued monthly is always fresh, therefore Indispensable to the practitioner who desires to keep posted up with the new inventions and improvements which are daily developed. Neither expense nor effort will be spared to give value and variety to its contents Articles will be illustrated with well executed engravings whenever they will add to the interest or assist to explanation.

Its extensive circulation and frequency of issue make it one of the BEST DENTAL ADVERTISING MEDIUMS in the United States.

All subscriptions must begin with January or July.

Local or special notices, five lines or less, will be inserted for $3 each insertion; over five lines, 50 cts. per line.

All advertising bills payable in advance, or at furthest, after the issue of the first number containing the advertisement. All transient advertisements to be paid for in advance.

☞CONTRIBUTIONS SOLICITED.

Exclusively Editorial Communications should be addressed to the Editor.

The latest number of the REGISTER may be ordered with or without other goods at any time, and all letters should be addressed to

**SPENCER & CROCKER, Ohio Dental & Surgical Depot, Cincinnati, O.**

